# Supplementary material for: Conditional chemoconnectomics (cCCTomics) as a strategy for efficient and conditional targeting of chemical transmission
Source: eLife. 2024 Apr 30;12:RP91927. doi: 10.7554/eLife.91927 (PMC11060718; doi:10.7554/eLife.91927)
Supplement: Figure 3—source data 3. [file elife-91927-fig3-data3.zip › Figure 3 source data3 part 1.zip!/Figure 3-Source data 3-part1/1082-9-CRISPResso_Report_zod2ewbs.zip!/CRISPResso_on_1082_10/3b.Insertion_deletion_substitutions_size_hist.pdf]

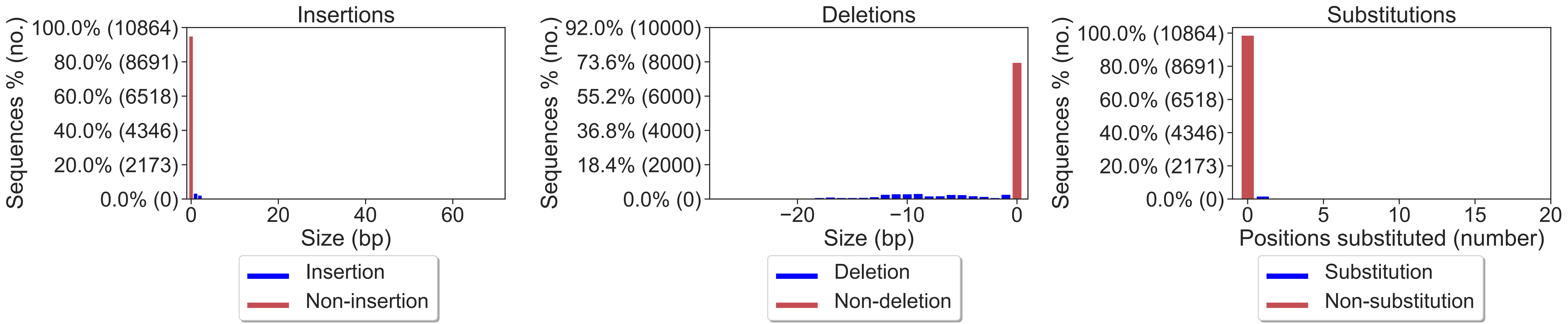

Insertions
Sequences % (no.)
100.0% (10864)
80.0% (8691)
60.0% (6518)
40.0% (4346)
20.0% (2173)
0.0% (0)
0
20
40
60
Size (bp)
Insertion
Non-insertion
Deletions
Sequences % (no.)
92.0% (10000)
73.6% (8000)
55.2% (6000)
36.8% (4000)
18.4% (2000)
0.0% (0)
−20
−10
0
Size (bp)
Deletion
Non-deletion
Substitutions
Sequences % (no.)
100.0% (10864)
80.0% (8691)
60.0% (6518)
40.0% (4346)
20.0% (2173)
0.0% (0)
0
5
10
15
20
Positions substituted (number)
Substitution
Non-substitution
